# American Dental Association

**Published:** 1886-08

**Authors:** 


THE AMERICAN DENTAL ASSOCIATION.—This Association held its 26th annual meeting at Niagara Falls, commencing August 3d, 1886, with the following "bit of facetiæ," in the presence of some two hundred members:

"Gentlemen, the hour has arrived when in my official capacity it becomes my pleasing duty to declare this convention formally opened," said Dr. W. C. Barrett, the president, as he advanced to the front o the Casino stage and raised his hand to silence the conversation in progress among the dentists assembled in the handsome auditorium.

"I call the president to order," rang out the sharp tones of Dr. George W. Keely, of Oxford, O.

The startled presiding officer glanced with a puzzled air of interrogation in the direction of the erect form of the treasurer, who stood with folded arms as calm as the Sphinx.

"No one has a right to call the president to order," continued Dr. Barrett after a long pause for explanation. "The gentleman from Oxford may rise to a point of order."

"I rise to a point of order," in the same cold, stern tones.

"State it," with fierce emphasis.

"According to the rules of this association no one has a right to put in his gab until he has paid his dues."

The presiding officer, who had been so busily engaged in other matters that he had forgotten the little financial formality, reddened, then joined heartily in the laugh at his expense, and quickly transferred a five-dollar greenback from his vest-pocket to the treasurer's drawer.

The twenty-sixth annual meeting of the American Dental Association was opened in the very town where over a quarter of a century ago the society was first ushered into being. Without exception every member present bore the unmistakable stamp of a gentleman. Calm, self-possessed and perfectly at ease, with that confident air which always characterizes those who approach the other members of their race from a vantage point, they formed a fine deliberative assembly, gathered from the four quarters of the United States to discuss the underlying principles and peculiar problems of their calling.

The exhibits of dental specialties and supplies included nearly everything pertaining to the preservation of the teeth. The Casino building was well adapted to this purpose, isasmuch as the main auditorium is nearly surrounded by wide corridors, music rooms and other apartments convenient of access and well lighted. The most elaborate display was made by the S. S. White Dental Manufacturing Company, of Philadelphia, said to be the largest establishment of the kind in the world. Upon four long tables were spread out bushels of artificial teeth properly classified, forceps in a hundred divers forms, drills, gouges, burs, burnishers and all the other instruments used in the filling process, the apparatus used in the manufacture, storage and administration of laughing gas, appliances for the manufacture of sets of artificial teeth, and in short everything pertaining to dental science. The company was represented by Dr. James W. White, the president, T. A. Long, the general traveling agent, and several assistants.

The second exhibit in point of size was that made by the Buffalo Dental Manufacturing Company, Dr. T. G. Lewis and Charles A. Rother in charge. They showed a complete line of dental goods, including many ingenious specialties which are handled exclusively by this company. The new asbestos heater shown on their table was much admired. A gas flame from a special form of cylindrical burner streamed up against a perpendicular fibrous surface of asbestos which is immediately transformed to a glowing mass which radiates the heat instead of permitting it to ascend in a current from the burning gas. This form of heater is excellent for a small operating room, while when set in a carved fireplace it forms an excellent substitute for a glowing grate.

The Florence Manufacturing Company, represented by G. A. Wells, exhibited some new forms of tooth brushes. The prophylactic brush by means of the curved handle, the tapered end and the contour of the bristles, will reach every exposed portion of the teeth as no other brush can, while the dental plate brush seems to be the only kind which will perfectly clean a plate. The other exhibitors were H. D. Justi, Philadelphia, artificial teeth and specialties; Seabury & Johnson, New York, dressings and absorbents; Welch Dental Company, Philadelphia, English artificial teeth; the Wilmington Dental Manufacturing Company, specialties; Gideon Sibley, Philadelphia, artificial teeth; American Dental Company, New York, dental specialties; Cutting & Delaney, Buffalo, carved woodwork, and C. A. Timme & Co., Hoboken, N. J., gold foil and cements for filling.

